# Eye position modulates retinotopic responses in early visual areas: a bias for the straight-ahead direction

**DOI:** 10.1007/s00429-014-0808-7

**Published:** 2014-06-19

**Authors:** Francesca Strappini, Sabrina Pitzalis, Abraham Z. Snyder, Mark P. McAvoy, Martin I. Sereno, Maurizio Corbetta, Gordon L. Shulman

**Affiliations:** 1 Department of Neurology, Washington University, School of Medicine, Saint Louis, MO 63110 USA; 2 Department of Psychology, Sapienza University of Rome, 00185 Rome, Italy; 3 Neuropsychology Research Centre, Scientific Institute for Research, Hospitalization, and Health Care (IRCCS, Istituto di Ricovero e Cura a Carattere Scientifico), Fondazione Santa Lucia, 00179 Rome, Italy; 4 Department of Motor, Human and Health Sciences, University of Rome “Foro Italico”, 00135 Rome, Italy; 5 Department of Radiology, Washington University, School of Medicine, Saint Louis, MO 63110 USA; 6 Birkbeck-UCL Centre for NeuroImaging, London, WC1H 0AP UK; 7 Department of Anatomy and Neurobiology, Washington University School of Medicine, Saint Louis, MO 63110 USA; 8 Neurobiology Department, Weizmann Institute of Science, 234 Herzl St., Rehovot, 7610001 Israel

**Keywords:** Gain field, Gaze, Retinotopy, Vertical meridian, Wide-field

## Abstract

Even though the eyes constantly change position, the location of a stimulus can be accurately represented by a population of neurons with retinotopic receptive fields modulated by eye position gain fields. Recent electrophysiological studies, however, indicate that eye position gain fields may serve an additional function since they have a non-uniform spatial distribution that increases the neural response to stimuli in the straight-ahead direction. We used functional magnetic resonance imaging and a wide-field stimulus display to determine whether gaze modulations in early human visual cortex enhance the blood-oxygenation-level dependent (BOLD) response to stimuli that are straight-ahead. Subjects viewed rotating polar angle wedge stimuli centered straight-ahead or vertically displaced by ±20° eccentricity. Gaze position did not affect the topography of polar phase-angle maps, confirming that coding was retinotopic, but did affect the amplitude of the BOLD response, consistent with a gain field. In agreement with recent electrophysiological studies, BOLD responses in V1 and V2 to a wedge stimulus at a fixed retinal locus decreased when the wedge location in head-centered coordinates was farther from the straight-ahead direction. We conclude that stimulus-evoked BOLD signals are modulated by a systematic, non-uniform distribution of eye-position gain fields.

**Electronic supplementary material:**

The online version of this article (doi:10.1007/s00429-014-0808-7) contains supplementary material, which is available to authorized users.

## Introduction

Stable perception of the world depends on the integration of sensory and motor information from retinal and “extraretinal” signals, which enable an accurate representation of stimulus location even as the eyes change position (Andersen et al. 1985). Studies in primates have shown that this representation may be computed through gain fields (cf. review of Salinas and Sejnowski 2001). The concept of gain fields was introduced by Andersen and Mountcastle (1983), who observed that changes in eye position did not change the location or shape of receptive fields of neurons in V7a and LIP, but modulated the rate of neural firing to stimuli at a fixed retinal locus. Since this initial work, neurons influenced by eye position have been found in many primate striate and extrastriate areas, including visual areas as early as V1 (Trotter et al. 1992; Guo and Li 1997; Dobbins et al. 1998; Trotter and Celebrini 1999; Rosenbluth and Allman 2002; Durand, et al. 2010). Evidence for overt shifting of receptive fields by extraretinal signals has been found in higher visual areas [retinotopic updating in LIP (Colby et al. 1996); head-centered updating in VIP (Duhamel et al. 1998)].

Previous research has supported the idea that neurons coding eye position are not topographically organized (i.e., neurons with a preference for a specific eye position are not located close one another in a specific part of the brain) and that, as a consequence, eye-position modulations are canceled out at the population level (Galletti and Battaglini 1989; Bremmer 2000). However, this concept has recently been challenged by studies (Durand et al. 2010; Anzai et al. 2011) that have shown that the spatial distribution of gain fields is non-uniform, increasing the neural response to stimuli in the straight-ahead direction. Because the straight-ahead direction is likely to be behaviorally relevant, this gain field bias may serve to prioritize events directly in front of the head and the body (Durand et al. 2010, 2012).

Few studies in humans have used functional magnetic resonance imaging (fMRI) to investigate eye position modulations in early visual areas and a clear spatial organization of these modulation has not been demonstrated (Deutschlander et al. 2005; Andersson et al. 2007; Williams and Smith 2010; Merriam et al. 2013). Williams and Smith (2010) and Deutschlander et al. (2005) found a modulation in visual cortex by eye position even in absence of visual stimuli. Andersson et al. (2007) used a quarter-field stimulation to study evoked responses in V1 and showed a stronger response when eyes and head were centrally aligned. Recently, Merriam et al. (2013) measured the BOLD response in early visual areas to rotating wedge stimuli presented at different fixation positions. They found that eye position modulated the amplitude but not the phase of the response at a voxel, consistent with both retinotopic coding and gain field modulation. Importantly, the BOLD responses in different voxels varied sufficiently across eye positions to allow classification of eye position, indicating that the distribution of gain fields across an early visual area such as V1 was not strictly uniform. However, it was unclear whether this distribution showed any consistent spatial structure, as suggested by monkey single unit studies reporting a preference for the straight-ahead direction (Durand et al. 2010, 2012). Moreover, while electrophysiological studies on primates have investigated eye position modulations across both the azimuth and elevation dimensions, in our knowledge, only one study in humans has investigated eye position modulation in the elevation dimension, even though across a limited range of eccentricities, ±5° (Merriam et al. 2013).

In the current study, we examined whether gaze modulations in early visual areas of humans reflected a bias for the straight-ahead direction along the elevation dimension. To answer this question we investigated the relation between gaze position (±20°) near the vertical meridian and blood oxygenation level dependent (BOLD) response to rotating polar angle wedges presented with a wide-field display set-up. We chose a phase-encoded paradigm because we originally out to examine whether gaze position alters the retinotopic positions of the receptive fields of neurons. We found no such position changes but instead discovered changes in response gain. The phase-encoded paradigm is less sensitive than a simple event-related or block paradigm would be for quantifying response gain but we found that sensitivity was nonetheless adequate and so we decided against performing new experiments.

## Materials and methods

### Overview

The experimental procedure included multiple fMRI sessions carried out in each subject. In aggregate, these sessions included retinotopic mapping, the main experiment in which gaze angle and retinotopic stimulation were varied, and a set of anatomical scans used for individual brain surface reconstruction.

### Participants

The subjects were six healthy adults with normal or corrected-to-normal visual acuity (mean age 27 years, range 26–31, 1 female), with no past history of psychiatric or neurological disease. All subjects had extensive experience in psychophysical and fMRI experiments and were paid for their participation. All participants gave written informed consent. All procedures were approved by the local Ethics and Human Subjects Committees. Subjects were allowed to consume caffeinated beverages before scanning to maintain alertness.

### Visual stimuli

#### Retinotopic mapping

We mapped responses to polar angle (measured from the contralateral horizontal meridian around the center of gaze) and eccentricity (distance from the center-of-gaze) using standard phase-encoded retinotopic stimuli (Sereno et al. 1995). The stimuli were presented using a wide-field display (Pitzalis et al. 2006) and consisted of high contrast light/dark colored checks flickering in counterphase at 8 Hz in either a wedge or a ring configuration (polar angle and eccentricity mapping, respectively) extending over 100° of visual angle (see “Experimental set-up” for details). The eccentricity ring expanded linearly with a uniform velocity ~1°/s. The average luminance of the stimuli was 105 cd/m^2^. The duration of one complete polar angle or eccentricity cycle was 64 s; 8 cycles were presented during each fMRI run. During retinotopic mapping, subjects were required only to maintain fixation on a central cross. This retinotopic mapping (polar angle and eccentricity) allowed us to define the boundaries of retinotopic cortical areas (V1, V2, V3, V3A, V7, VP, V4v and V4/V8) on the cortical surface for each individual subject on the basis of the visual field sign (Sereno et al. 1995; see “Data analyses” for details).

#### Gain field experiment: interaction between gaze position and retinotopy

**Fig. 1:**
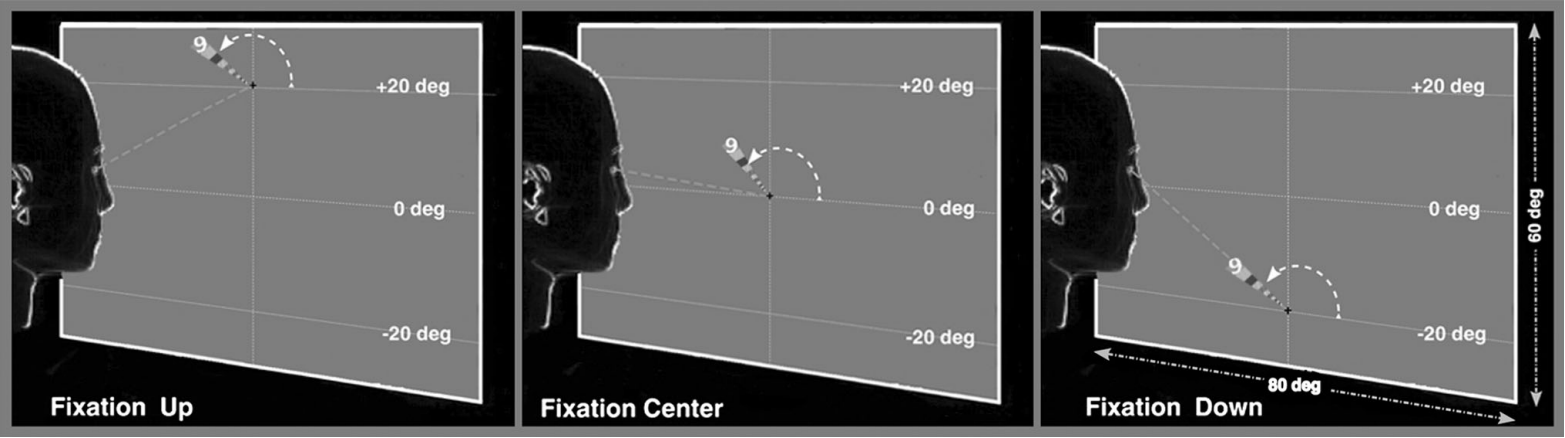
Design of the Gain Field Experiment. The three screens represent three different gaze position conditions (+20°, 0°, −20° vertical), performed separately in different fMRI runs. Visual stimulation consisted of a flickering checkerboard wedge rotating in a counterclockwise direction, subtending 10°. Three subjects performed a covert visual attention task (letterotopy), which involved detecting a digit as opposed to letters presented along the wedge

In the same group of subjects, we performed an additional retinotopic experiment that tested the interaction between gaze position and retinotopy (i.e., gain field effect). In this study (hereafter designated the *gain field experiment*), during separate scans we presented 10° radius rotating wedge stimuli centered either straight ahead (in the head-centered coordinates) or vertically displaced by ±20° (Fig. 1). Thus, in the three gaze-conditions (gaze-up, gaze-center, and gaze-down), the stimulated screen locations were completely non-overlapping. These stimuli were presented using a wide-field display; however, here the polar angle stimulus was small, extending up to ±20° as in the majority of the fMRI experiments (see Fig. 1). In all conditions, subjects maintained fixation on a crosshair subtending about 0.5° as the wedge rotated about the center of the gaze at 1° of eccentricity (0.5° of space between the fixation cross and the beginning of the stimulus.) Three out of the subjects passively viewed the checkerboard wedges during the scans (hereafter designated the *passive* *gain field*). The other three subjects performed a task that encouraged covert visual attention to the wedge (hereafter designated the *letterotopy* experiment or *attentional* gain field). This task was chosen based on several fMRI studies showing that BOLD responses can be modulated by attentional mechanisms in areas as early as V1 (Brefczynski and DeYoe 1999; Kastner et al. 1999; Somers et al. 1999; Sereno and Amador 2006; Saygin et al. 2004; Saygin and Sereno 2008). In the attentional gain field experiment, the wedge contained superimposed stream (2.85 Hz, asynchronous) of eccentricity-scaled letters. Subjects were required to fixate on the center cross while monitoring for occasional number (amongst letters, see Fig. 1), which were rare events (5 % of trials). Subjects were asked to mentally count how many digits appeared during each letterotopy run and to verbally report this count at the end of fMRI run. Compared to plain checkerboards, the additional visual tasks have been found to more consistently activate both lower and higher visual areas in humans (e.g., Sereno et al. 2001; Pitzalis et al. 2006, 2010, 2013).

## Experimental set-up

Visual stimuli were generated using an in-house X11/OpenGL program (original GL code by A. Dale, supported and extended by M. Sereno; Mapper software: http://kamares.ucsd.edu/~sereno/stim/) and a Tiga-diamond (Salient AT3000) graphics card. An LCD video projector (Sharp GX-3800, 640*480 pixels, 60 Hz refresh) with a customized lens projected stimuli onto a back-projection screen attached to the back of the head coil. Head position was stabilized with foam padding. For both experiments we used a wide-field set-up similar to that previously described (Pitzalis et al. 2006). To get a wide-field stimulation, we lowered the subject’s body by about 4 cm from iso-center so that the bottom portion of the screen was not blocked and we used an enlarged mirror so that the screen periphery was visible. The size of the screen subtended up to 100° (±50°) horizontally, 80° (±40°) vertically, and 110° (±55°) in an oblique direction. The eye-to-screen light path was about 18 cm. At this short viewing distance, visual stimuli for the retinotopic mapping subtended up to 100° (±50°) horizontally and 80° (±40°) vertically; visual stimuli for the gain field experiment subtended up to 80° (±40°) horizontally and 60° (±30°) vertically. Besides enabling wide-angle stimuli, this arrangement also helped to control a critical confound in fMRI mapping studies caused by surround inhibition (Brewer et al. 2002). As previously explained (Sereno and Tootell 2005; Pitzalis et al. 2006, 2010, 2013), retinotopic cortical regions with representations of visual space just beyond the peripheral edge of a rotating wedge can generate misleading 180° out-of-phase periodic response. The wide-field arrangement greatly reduces this confound.

### Imaging parameters

The fMRI experiments were conducted at the Santa Lucia Foundation (Rome, Italy) using a 3T Allegra scanner (Siemens Medical Systems, Erlangen, Germany). Single-shot echo-planar imaging (EPI) images were acquired with interleaved slice ordering using a standard transmit-receive birdcage head coil. For wide-field retinotopic mapping, 30 slices (2.5 mm thick, no gap, in-plane resolution 3 × 3 mm) perpendicular to the calcarine sulcus were collected. Each participant underwent four consecutive scans (two polar angle and two eccentricity). To increase the signal to noise ratio, data were averaged over two scans for each stimulus type (eccentricity and polar angle).

For the gain field experiment 30 slices (3.5 mm thick, no gap, in-plane resolution 3 × 3 mm) parallel to the anterior-posterior commissural plane were collected: 3.5 mm thick (no gap, interleaved excitation order), with an in-plane resolution of 3 × 3 mm. The gain field experiment was conducted on two separate days. Each day included six fMRI runs of polar angle stimulus covering all gaze positions (two runs with central fixation, two runs with upper fixation, and two runs with the lower fixation) for a total of 12 runs over both days. Within each run eye position was held constant. Eye position order varied randomly across runs, sessions and subjects. In both experiments, each run included 256 single-shot EPI images per slice [repetition time (TR), 2,000 ms; echo time (TE) 30 ms, flip angle 70°, 64 × 64 matrix; bandwidth 2,298 Hz/pixel; FOV 192 × 192 mm]. Overall, 16 fMRI runs were carried out in each of the 6 subjects (4 runs of retinotopy plus 12 runs for the gain field experiment) for a total of 96 fMRI runs.

The cortical surface of each subject was reconstructed from 3 structural scans (T1-weighted sagittal Magnetization Prepared Rapid Gradient Echo (MPRAGE) sequence, TI = 910 ms, TE = 4.38 ms, flip angle = 8°, 256 × 256 × 176 matrix, 1 mm^3^ voxels, bandwidth = 130 Hz/pixel). At the end of each session, an MPRAGE alignment scan was acquired parallel to the plane of the functional scans. The alignment scan was used to establish an initial registration of the functional data with the brain surface. Additional affine transformations that included a small amount of shear were then applied to the functional scans using blink comparison with the structural images to achieve an exact overlay of the functional data onto each cortical surface.

### Data analyses

#### Anatomical image processing

FreeSurfer was used for surface reconstruction (Dale et al. 1999; Fischl et al. 1999). Briefly, the three high-resolution structural images, obtained from each subject, were manually registered and averaged. The skull was stripped off by expanding a stiff deformable template out to the dura, the gray/white matter boundary was estimated with a region-growing method, and the result was tessellated to generate a surface that was refined against the MRI data with a deformable template algorithm. By choosing a surface near the gray/white matter border (rather than near the pial surface, where the macrovascular artifact is maximal), we were able to assign activations more accurately to the correct bank of a sulcus. The surface was then unfolded by reducing curvature while minimizing distortion in all other local metric properties. Each hemisphere was then completely flattened using five relaxation cuts: one cut along the calcarine fissure, three equally spaced radial cuts on the medial surface, and one sagittal cut around the temporal lobe.

#### Analysis on the phase of the retinotopic signal: Fourier analysis

Retinotopic data from both experiments (wide-field retinotopic mapping and gain field) were analyzed using UCSD/UCL FreeSurfer (Dale et al. 1999; Fischl et al. 1999) based on standard procedures described in details in many previous publications (e.g., Sereno et al. 1995; Tootell et al. 1997; Hagler and Sereno 2006; Pitzalis et al. 2006, 2010, 2013). The first (pre-magnetization steady-state) four volumes were discarded. Motion correction and cross-scan alignment were performed using the AFNI (Analysis of Functional NeuroImages) 3dvolreg (3T data). Phase-encoded retinotopic data were analyzed by voxelwise Fourier transforming the fMRI time series (after removing constant and linear terms).

This Fourier analysis generates real and imaginary components (equivalently, amplitude and phase) at each frequency. To estimate the significance of the BOLD signal modulation at the stimulus frequency (eight cycles per scan), the squared Fourier amplitude was divided by the summed mean squared amplitude (power) at all other frequencies, which includes noise. The ratio of two Chi squared variates follows the *F*-distribution (Larsen and Marx 1986), with degrees of freedom equal to the number of time points from which statistical significance can be calculated. The second harmonic of the stimulus frequency and very low frequencies (1 and 2 cycles per scan, residual motion artifacts) were ignored. Response phase at the stimulus frequency was used to map retinotopic coordinates (polar angle or eccentricity). In these maps, hue represents phase and saturation represents a sigmoid function of the response amplitude. The sigmoid function was arranged so that visibly saturated colors begin to emerge from the gray background at a threshold of *p* < 10^−2^. Computed significance at the most activated cortical surface loci ranged from *p* < 10^−5^ to 10^−10^. Since this analysis does not take into account fMRI time series autocorrelation (Zarahn et al. 1997), these *p* values are properly regarded as descriptive. Boundaries of retinotopic cortical areas were defined on the cortical surface for each individual on the basis of phase-encoded wide-field retinotopy (DeYoe et al. 1994, 1996; Engel et al. 1994, 1997; Sereno et al. 1995) and subsequent calculation of visual field sign. This latter provides an objective means of drawing borders between areas based on the angle between the gradients (directions of fastest rate of change) in the polar angle and eccentricity with respect to the cortical surface (Sereno et al. 1994, 1995). Each field sign map used here was based on at least four scans (two scans for polar angle and two scans for eccentricity).

#### Defining retinotopic visual regions of interest (ROIs)

**Fig. 2:**
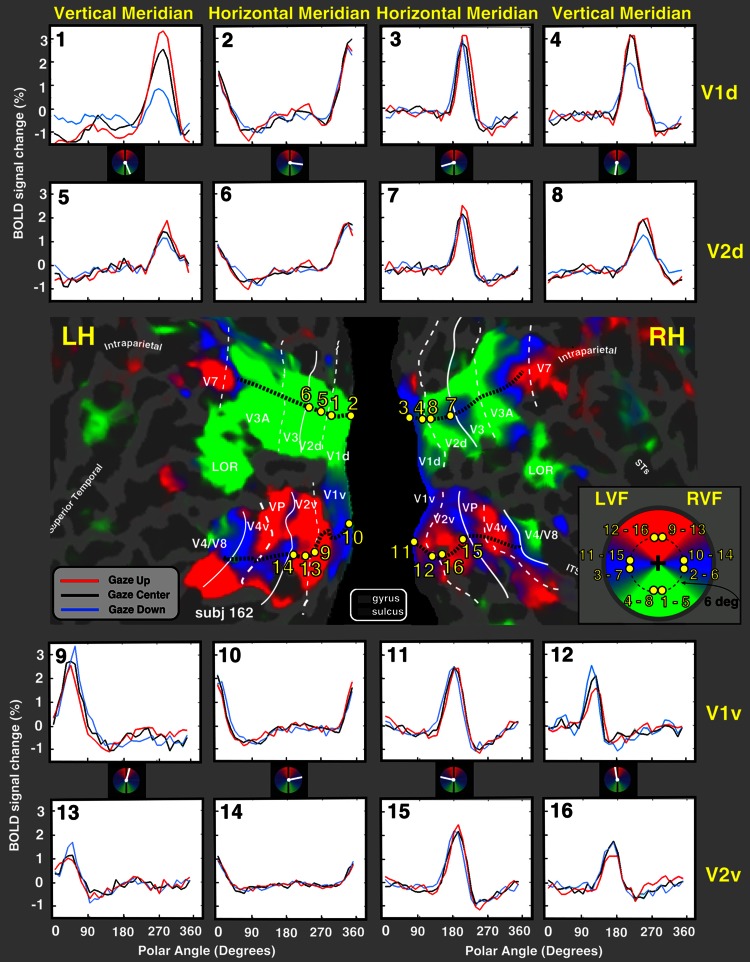
Gain field effect in a representative participant. The figure center shows a flattened representation of the posterior portion of the left and right hemispheres in a representative subject, overlaid with a polar angle map derived from all available data for this subject (12 runs of passive Gain Field Experiment). The *white lines* on the surface show the borders between the retinotopic visual areas. The *dotted* and *solid lines* indicate vertical and horizontal meridians, respectively. The *red*, *blue* and *green* areas represent upper, middle, and lower visual fields respectively. *Yellow points* on the surface indicate the (single-voxel size) regions of interest (ROIs), selected from the phase of the eccentricity and polar angle wide-field retinotopic maps. The inset polar plot (*right* of the figure) shows the distribution of the locations across the visual field corresponding to the sampled ROIs. On the individual surface of each subject we sampled 16 ROIs, at about 6° of eccentricity, in visual areas V1 and V2, close to the horizontal meridian (ROIs 2, 3, 6, 7, 10, 11, 14, 15) and the vertical meridian (ROIs 1, 4, 5, 8, 9, 12, 13, 16). Locations in the upper visual field correspond to ROIs in the ventral V1 and V2, whereas locations in the lower visual field correspond to ROIs in the dorsal V1 and V2. BOLD response time courses were extracted from these 16 ROI in every subject. The 16 graphs shown in the *upper* and *lower* parts of the figure show, respectively the response time courses extracted from the ROIs in V1d (1–4), V2d (5–8) and V1v (9–12), V2v (13–16). Each graph shows the response time courses of a single ROI for the three eye positions as a function of polar angle. The *black*, *red* and *blue lines* represent gaze-center, gaze-up and gaze-down condition, respectively. For each time point and time course the standard error of the mean was always <0.25, i.e., less than the width of the *plotted line*. Major sulci (*dark grey*) are labeled as follows: Intraparietal sulcus, STs (Superior Temporal sulcus); LOR (Lateral Occipital Region)

The wide-field retinotopic mapping was used here also to define in each individual subject subregions in visual areas V1 and V2. Specifically, for each subject (*N* = 6) sixteen single-voxel regions of interests (ROIs) were defined based on the analysis of phase-encoded polar angle data (Fig. 2). These ROIs comprised four loci in visual areas V1 dorsal (LH 1–2; RH 3–4), V2 dorsal (LH 5–6; RH 7–8), V1 ventral (LH 9–10; RH 11–12), and V2 ventral (LH 13–14; RH 15–16). For each visual area (e.g., V1 dorsal), loci were selected at 6° eccentricity, two close to the horizontal meridian (ROI 2–3) and two close to the vertical meridian (ROI—1–4; Fig. 2, middle panel). This eccentricity corresponds to the approximate center of the retinotopy wedges (which subtended 1°–10°). To exactly define isoeccentricity ROIs in the individual surface, we used the analysis of the eccentricity movie to reveal the eccentricity progression inside a specific cortical area, and to define the isoeccentricity band corresponding to 6° (Fig. 2, see logo in the middle panel, bottom right). Although a series of color maps with superimposed iso-eccentricity contour lines contains no more information than a single color map, the dynamic display enhances the perception of small but significant variations in eccentricity that are hard to see in static displays (see e.g., Hadjikhani et al. 1998; Pitzalis et al. 2006, 2010, 2013). To exactly define ROIs close to the horizontal and vertical meridian in the individual surface, we used the analysis of the polar angle movie to reveal the progression of the phase inside a particular cortical area.

Every retinotopic map was plotted on a flattened version of each participant’s reference anatomical cortical surface. Surface-defined ROIs were embedded into each subject’s volumetric fMRI data (projected outward by 2 mm from the gray-white boundary) using a custom procedure that linearly transformed FreeSurfer vertex coordinates into locations in 3D volumes. Each region was single-voxel size. Then BOLD time series were extracted from four 6° eccentric loci in each visual area (four fMRI runs (32 cycles) at each gaze condition in each subject). For display purposes, individual retinotopic ROIs were then projected onto the polar angle flat maps derived from the gain field experiment of each subject.

#### Analysis on the amplitude of the retinotopic signal: Time course and voxel-wise analysis

For each individual, the AFNI-preprocessed data were coregistered across sessions and then registered (12-parameter affine transform) to Talairach space using an atlas-representative template conforming to the SN method of Lancaster et al. (1995). After composition of transforms, the functional data were resampled in one step to 3 mm isotropic voxels. Voxelwise responses to polar angle modulations were extracted independently for each time point (32 frames per cycle) and gaze condition using a general linear model (GLM) (Friston et al. 1995; Ollinger et al. 2001). The GLM included nuisance regressors representing baseline, linear trend and low frequency components (<0.009 Hz). The resulting response (beta) maps were spatially smoothed (6 mm FWHM in each direction) and analyzed in single-subject and group ANOVAs. To assess statistical significance, non-independence of time points was taken into account by appropriately adjusting the degrees of freedom. Computed F-statistics were converted to equi-probable *Z* scores and significant responses were identified using joint *Z*-score/cluster size thresholds (*Z* > 3.0 over at least 13 face-contiguous voxels) (Forman et al. 1995).

**Fig. 3:**
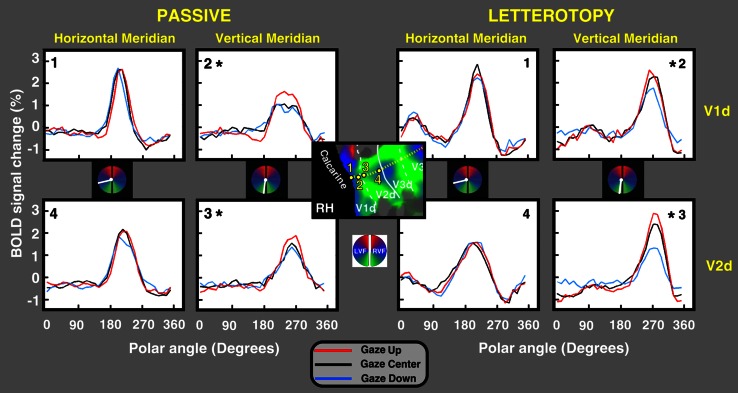
Polar angle responses, during gaze-up, gaze-center, and gaze-down conditions, during passive and attentional (letterotopy) gain field experiments. In the center is a close-up of the flattened representation of the right dorsal stream in occipital cortex of one representative subject, overlaid with a polar angle map derived from the average of all 12 scans. The *left* and *right* parts of the figure show the time courses for the eye positions (gaze-up, gaze-center and gaze-down) as a function of polar angle. The *left* and *right* panels show, respectively the average time courses from the passive and attentional (letterotopy) gain field experiments. The polar angle color-code and symbol conventions are as in Fig. 2. The *asterisk* indicates a significant (*p < 0.05, Bonferroni corrected) interaction between polar angle and gaze position

**Fig. 4:**
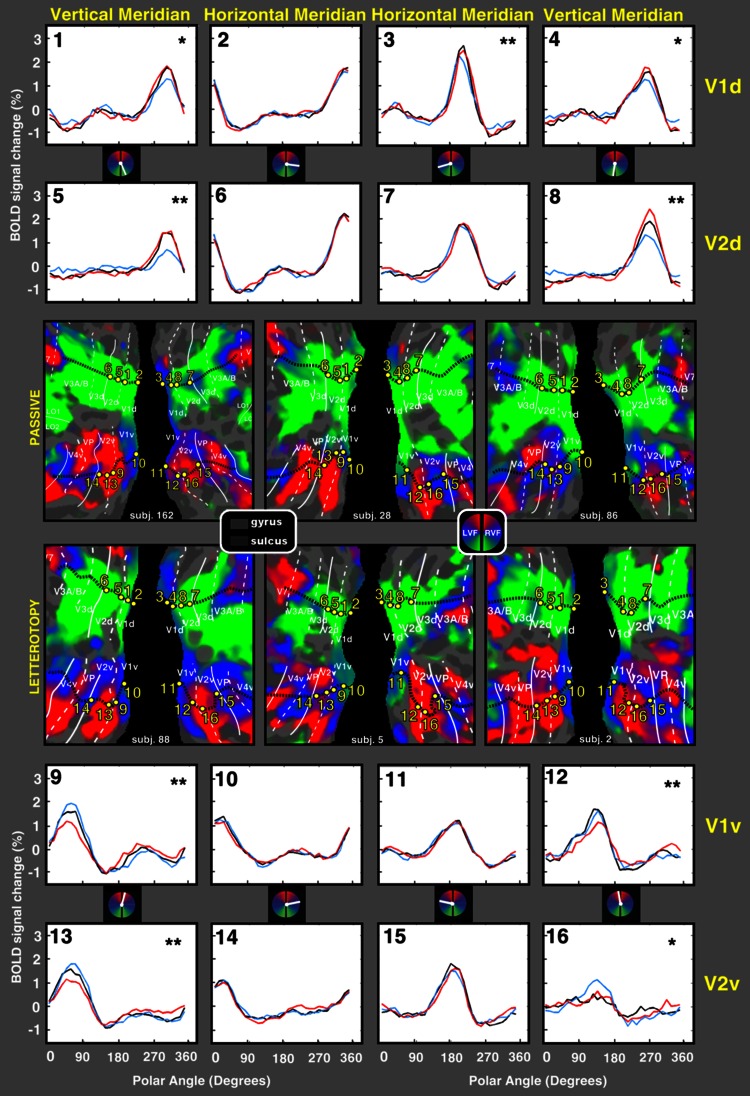
Averaged polar angle responses, during gaze-up, gaze-center, and gaze-down conditions, across all six subjects. In the center, the flattened representations of the right and left occipital cortices of all six participants, overlaid with a polar angle map derived from all available gain field data (passive, *top row*; letterotopy, *bottom row*). The polar angle color-code and symbol conventions are as in previous figures. *Asterisks* indicate significant (*p < 0.05, **p < 0.001 Bonferroni corrected) interaction between polar angle and gaze position

To study the interaction between gaze position and polar angle in the gain field experiment, we performed a series of analyses. First, we conducted two group-level ANOVAs treating subjects as a random effect, and using single-voxel retinotopic regions-of-interest (ROIs) drawn on the individual surfaces of each subject (Figs. 3, 4). The first group-level ANOVA (Fig. 3) was performed to assess differences between passive and letterotopy condition, thus we analyzed only the two more extreme gaze positions (up and down) in order to study any qualitative differential trend. This ANOVA (Fig. 3) included three within-subject factors: gaze position (2 levels: up and down), polar angle (32 levels corresponding to polar angle during 32 volumes), and meridian (2 levels: horizontal and vertical, responses assessed over several ROIs); the letterotopy and passive retinotopy conditions were analyzed independently. The second group-level ANOVA (Fig. 4) was performed to specifically asses the gain field effect. This second analysis was identical to the first, except that the gaze factor included three levels (center, up, and down); letterotopy and passive retinotopy conditions were analyzed jointly.

**Fig. 5:**
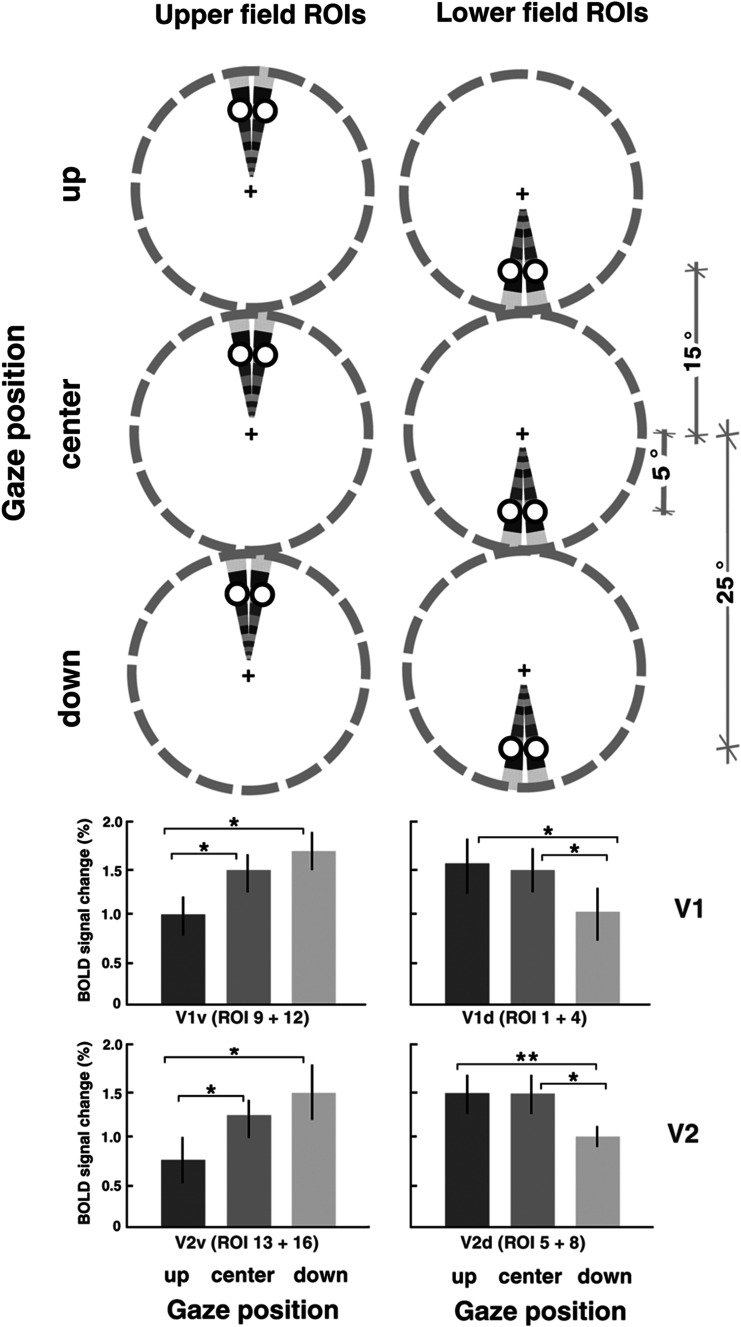
Averaged response amplitude during gaze-up, gaze-center, and gaze-down conditions on the peak response, across all six subjects. The vertically aligned *dashed circles* in each column represent the three different gaze position conditions (−20°, 0°, +20° vertical). Visual stimulation consisted of a flickering checkerboard wedge rotating in a counterclockwise direction. *White points* on the wedges indicate the (single-voxel size) regions of interest. For each visual cortical area four regions have been sampled near the vertical meridian (V1: region 1, 4, 9, 12; V2: region 5, 8, 13, 16; see Fig. 2 for further ROIs details). *Graph bars* indicate BOLD signal change in the gaze-up, gaze-center, and gaze-down conditions in cortical visual areas V1 and V2. The two panels represent the set of points sampled close to the vertical meridian in the upper and lower visual field, respectively. *Error bars* represent ± SEM (*p < 0.05)

**Fig. 6:**
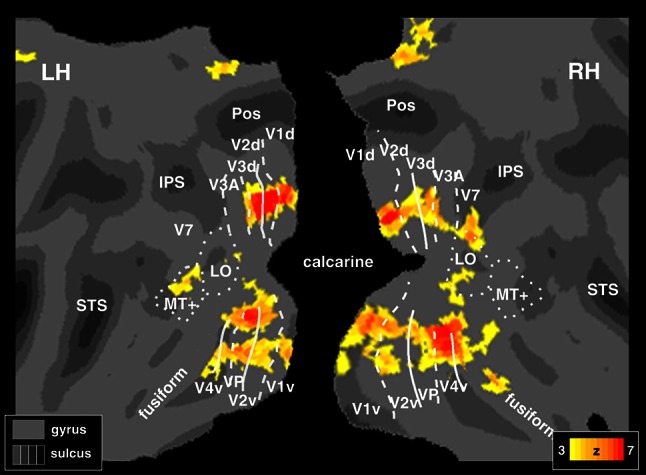
Interaction between gaze position and polar angle. The interaction was computed at the group level, plotted on the flattened representations of the right and left occipital cortices using Caret software (Van Essen 2005). The *white lines* show the borders between the retinotopic visual areas: the *dotted* and *solid lines* indicate vertical and horizontal meridian, respectively

**Fig. 7:**
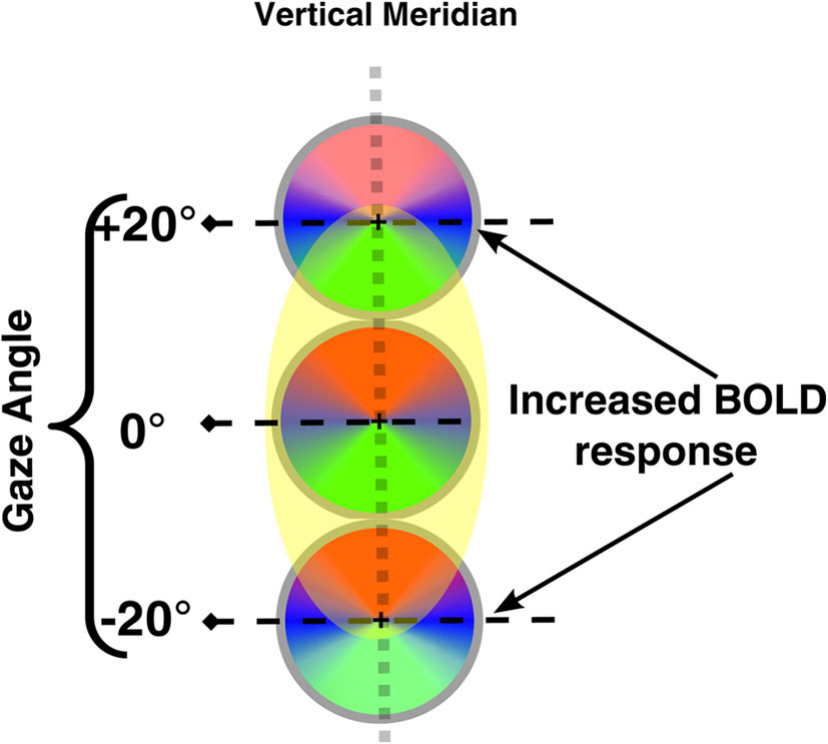
Schematic representation of the gain-field effect. BOLD responses are increased for central positions of the visual field (head-centered coordinates). Conversely, responses for lower positions are attenuated in the gaze-down condition (−20°) as well as for upper positions in the gaze-up condition (+20°)

Second, we conducted a similar ANOVA and *t* tests based on the magnitude of the peak BOLD response rather than on the entire set of 32 polar angles. The magnitude ANOVA (Fig. 5) included two within-subjects factors: gaze position (3 levels, up/center/down) and visual field location (2 levels, up/down), separately conducted for both visual areas V1 and V2. For each ROI we averaged the time series from each subject and then we estimated the amplitude by averaging ± 1 time points around the peak (Figs. 4, 5, 6, 7, Supplementary Figures 8, 9).

Third, we also conducted a voxel-wise group level ANOVA (Fig. 6) to assess the spatial topography of gaze modulations not only in V1 and V2 but also across all early visual areas, as individually defined by the wide-field retinotopic mapping. This ANOVA (Fig. 6) included two factors: gaze position (center, up and down) and polar angle (32 levels as above). Significance of the voxel-wise gaze-position × polar angle interaction map was assessed using cluster-based Monte Carlo-derived *Z*-score and extent thresholds (McAvoy et al. 2001).

## Results

The goal of this study was to characterize the spatial distribution of gain field modulations by eye position in early visual areas near the vertical meridian. Rotating flickering checkerboard wedges were presented at three positions on the screen (+20°, 0°, and −20° of eccentricity) in separate scans (Fig. 1). To improve activation and signal-to-noise ratios, three subjects performed a task that required covert visual attention to the wedge (attentional gain field). Subjects mentally counted how many numbers appeared during the visual stimulation and verbally reported their count at the end of each scan. The average accuracy was 93 %, indicating that subjects performed this continuous task appropriately. The other three subjects passively viewed similar checkerboard wedges (passive gain field).

To study the BOLD fMRI response to these stimuli, we identified the borders between the early visual areas with standard retinotopic mapping methods and wide-field retinotopic stimulation that has been described previously (Sereno et al. 1995; Pitzalis et al. 2006). For left and right dorsal and ventral V1 and V2 in each subject, we defined one ROI bordering the horizontal meridian and one ROI bordering the vertical meridian. Each ROI was located at about 6° of eccentricity, the approximate center of the checkerboard stimuli (Fig. 2, see caption for details). In order to see if the spatial distribution of eye position modulations resulted in increased responses for locations nearer the straight-ahead direction, we examined the BOLD response of each of these regions in each subject. If a straight-ahead bias is present, BOLD responses should be enhanced for wedges located in the central part of the visual field relative to the head.

### Time course is modulated by eye position: individual results

We found a consistent pattern in both V1 and V2: regions near the vertical meridian showed an effect of gaze position as a function of the polar angle that was consistent with a gain modulation (Andersen and Mountcastle 1983). Moreover, the BOLD response to a wedge at a fixed retinotopic location along the vertical meridian was enhanced for gaze conditions that positioned that location nearer to the straight-ahead direction (in head coordinates). Regions near the horizontal meridian, by contrast, seemed not to be affected by the gaze position as a function of the polar angle. Representative time courses from a single subject are shown in Fig. 2. A qualitative description of the figure suggests that both V1 and V2 showed a gain modulation only for locations near the vertical meridian. In particular, in V1 dorsal and V2 dorsal, responses for lower field positions were attenuated in the gaze-down condition compared to the gaze-center and gaze-up conditions (Fig. 2, time courses 1, 4, 5, 8). Conversely, in V1 ventral and V2 ventral, the time courses showed the opposite trend (Fig. 2, time courses 9, 12, 13, 16). In this case, the response for upper field positions was decreased in the gaze-up condition in comparison to gaze-center and gaze-down conditions. The observed response attenuation when stimuli were positioned most eccentrically with respect to the head suggests a preference for the straight-ahead direction, i.e., a bias toward central stimuli in body-centric coordinates.

### No qualitative difference between passive and letterotopy condition

Responses to passive and letterotopy stimuli were qualitatively similar: both groups showed a gain field effect with a response bias for the straight-ahead direction (Fig. 3). Post-hoc tests showed that gaze up/down × polar angle interactions were significant along the vertical meridian but not along the horizontal meridian. Comparable statistical significance was obtained in both V1 and V2 and in the passive and letterotopy conditions. Figure 3 shows the results for a representative region, the right dorsal visual occipital cortex, for passive viewing (V1 dorsal, vertical meridian, region 2, *F*(32, 128) = 1.872, *p* < 0.05; V2 dorsal, vertical meridian, region 3, *F*(32, 128) = 1.716, *p* < 0.05) and letterotopy conditions (V1 dorsal, vertical meridian, region 2, *F*(32, 128) = 1.819, *p* < 0.05; V2 dorsal, vertical meridian, region 3, *F*(32, 128) = 2.451, *p* < 0.05). Since the two groups did not qualitatively differ in the gain field effect, they were collapsed in subsequent analyses.

### Time course analyses: group results

We tested the statistical significance of the results by performing a group ANOVA with the factors gaze position (up/center/down), polar angle (32 levels), and meridian (horizontal/vertical), and treating subjects as a random effect (Fig. 4, see caption for details). Figure 4 shows the average time course across all six subjects, extracted from the individually-defined ROIs in each subject. In Fig. 4 meridians were defined using the same individual ROIs identified above (see Fig. 2). The group-level ANOVA yielded a significant three-way interaction between gaze position, polar angle, and meridian in all eight areas (left and right, dorsal and ventral V1 and V2; *F*(62, 480) > 1.43; *p* < 0.05, Bonferroni corrected). Post-hoc tests showed that up/center/down × polar angle interactions were significant along the vertical meridian (V1 dorsal, right hemisphere, vertical meridian, region 4, *F*(62, 480) = 1.370, *p* < 0.05; V2 dorsal, right hemisphere, vertical meridian, region 8, *F*(62, 480) = 3.493, *p* < 0.001, V1 ventral, right hemisphere, vertical meridian, region 12, *F*(62, 480) = 2.149, *p* < 0.001, V2 ventral, right hemisphere, vertical meridian, region 16, *F*(62, 480) = 1.430, *p* < 0.05; V1 dorsal, left hemisphere, vertical meridian, region 1, *F*(62, 480) = 1.859, *p* < 0.05; V2 dorsal, left hemisphere, vertical meridian, region 5, *F*(62, 480) = 5.193, *p* < 0.001, V1 ventral, left hemisphere, vertical meridian, region 9, *F*(62, 480) = 2.455, *p* < 0.001, V2 ventral, left hemisphere, vertical meridian, region 13, *F*(62, 480) = 2.004, *p* < 0.001) but not along the horizontal meridian in all areas, with one exception: the right region in V1 dorsal (in the right hemisphere) along the horizontal meridian also showed a significant effect (region 3, *F*(62, 480) = 2.455, *p* < 0.001). The results match what was observed in the individuals: enhanced response to wedges in gaze conditions that positioned the wedge nearer the straight-ahead direction (in head-centered coordinates).

### Response amplitude analyses: gaze modulations on the peak response

Because the ANOVA included all 32 polar angles as levels, the significant effects of the polar angle factor could have reflected subtle eye position modulations over a range of polar angles rather than at the polar angle yielding the peak BOLD response. Therefore, we also conducted analyses that specifically looked at the effects of gaze condition on the peak response. The peak BOLD amplitude in a certain condition was estimated by averaging the amplitudes of the 3 MR frames that were centered on the frame that yielded the peak amplitude in the group (after averaging over gaze conditions to avoid a bias in frame selection). We directly compared fMRI response amplitudes in gaze-up, gaze-center, and gaze-down condition at the same retinotopic ROIs, shown as the white disks in Fig. 5 (regions 9, 12, 13, 16 for the upper visual field, regions 1, 4, 5, 8 for the lower visual field, see Fig. 2 for details on the ROIs). These locations are retinotopically identical (being all at 6° of constant distance from their relative fixation point), but they are not at the same distance from the straight-ahead direction (gaze-center). Indeed, the distance from straight-ahead is 26° in the gaze-up (V1–V2 ventral) and gaze-down conditions (V1–V2 dorsal), 14° in the gaze-down (V1–V2 ventral) and gaze-up (V1–V2 dorsal) conditions, 6° for the gaze-center (V1–V2 ventral and dorsal). We performed a two-way repeated-measures ANOVA with gaze position (up/center/down) and visual field location (upper/lower) as factors, separately conducted for both visual area V1 and V2. The ANOVA showed a significant interaction between the two factors in both V1 and V2 (V1: *F*(2,5) = 17.041, *p* = 0.001; V2: *F*(2,5) = 32.179, *p* = 0.0001) but no other effects. Separate paired *t* tests were then conducted comparing gaze-up, gaze-center, and gaze-down conditions for the upper field ROIs (left panel, Fig. 5) and lower field ROIs (right panel, Fig. 5). For the upper-field ROIs, response amplitude was significantly higher in the gaze-down and gaze-center than gaze-up conditions in V1 ventral and V2 ventral (Fig. 5, left panel, V1 ventral gaze-down vs gaze-up: *t*(5) = 4.58, *p* = 0.005; V1 ventral gaze-center vs gaze-up: *t*(5) = 3.57, *p* = 0.016; V2 ventral gaze-down vs gaze-up: *t*(5) = 6.88, *p* = 0.0009; V2 ventral gaze-center vs gaze-up: *t*(5) = 2.63, *p* = 0.04); conversely, for the lower-field ROIs, response amplitude was significantly higher in the gaze-up and gaze-center than gaze-down conditions in V1 and V2 dorsal (Fig. 5, right panel, V1 dorsal gaze-up vs gaze-down: *t*(5) = 3.09, *p* = 0.027; V1 dorsal gaze-center vs gaze-down: *t*(5) = 4.22, *p* = 0.008; V2 dorsal gaze-up vs gaze-down : *t*(5) = 3.51, *p* = 0.017; V2 dorsal gaze-center vs gaze-down : *t*(5) = 2.94, *p* = 0.03). Overall, BOLD response amplitude was significantly higher for a fixed retinotopic location near the vertical meridian when the gaze direction positioned that location nearer the straight-ahead direction (regions at 6° and 14° of distance from straight-ahead direction).

As a control, we repeated the analysis with amplitudes derived from a GLM. For each subject, each condition was modeled using a separate regressor in the GLM. The regressor was created by convolving a stimulus function with an assumed hemodynamic response function (HRF), where the function was shifted based on the phase determined from the fourier analysis of the polar angle scans. We found that the effect does not change near the vertical meridian (see Supplementary Fig. 8). Therefore, the ANOVA on the peak response and derived from the GLM confirmed the results obtained with the previous ANOVA (Fig. 4), which was conducted using all 32 levels of the polar angle variable.

### Topographic distribution of the interaction between polar angle and eye position

The above results concerned visual areas V1 and V2. Extending the analyses beyond V1 and V2, at the group level, revealed significant gaze × polar angle interactions in all early visual areas, particularly between 5° and 10° of eccentricity along the vertical meridian (Fig. 6). However, this interaction was significant also along the horizontal meridian, possibly because of imperfect registration of visual areas across subjects in a group analysis. These results indicate that enhanced responses to the straight-head direction, as indexed by the interaction between gaze and polar position, might be present in all early visual areas.

### Phase maps in retinotopic areas do not change with gaze position

Many studies in monkeys and humans show that gaze position changes the response gain of neurons, but not the retinotopic position of their receptive fields (Zipser and Andersen 1988; Chang et al. 2009; DeSouza et al. 2002; Siegel et al. 2006; Merriam et al. 2013). Supplementary Fig. 9 shows phase maps from the polar angle scans for gaze-up, gaze-center, and gaze-down conditions in two subjects. The topography of the phase maps from the wedge (i.e. polar angle) scans did not systematically change with eye position, consistent with retinotopic coding. The constancy of the phase angle map, shown here qualitatively, has recently been demonstrated in detail by Merriam et al. (2013).

## Discussion

While many studies have investigated eye position gain fields and their importance in spatial localization, less is known about their role in visual processing. The aim of this study was to test the null hypothesis that gain field modulations are uniformly distributed across early visual areas in human cortex. Our results provide evidence of enhanced responses to stimuli nearer the straight-ahead direction, consistent with recent findings in monkeys (Durand et al. 2010), but also indicate that gaze-dependent modulations are not solely governed by the distance of the stimulus from straight-ahead.

In the present study we focused the data collecting on the elevation dimension. Most of the fMRI studies on eye position have investigated only the azimuth dimension. Hence, wide-field display was an ideal set-up for investigating eye modulations along the elevation dimension for the first time over a wide range of visual eccentricities.

### Gaze modulations increase the priority of locations nearer to straight-ahead

Gaze modulations we observed are consistent with recent proposals that response amplitudes of peripheral neuron in V1 are increased for retinotopic locations nearer the straight-ahead direction (Durand et al. 2010). A schematic representation of this result is presented in Fig. 7. The BOLD response evoked by a wedge at a retinotopically fixed upper-field location was reduced when subjects fixated above vs. center/below the straight-ahead direction. Conversely, the BOLD response evoked by a wedge at a retinotopically fixed lower-field location was reduced when subjects fixated below vs. center/above the straight-ahead direction. This effect was consistent across subjects and was present in cortical regions representing a wide range of visual field-eccentricities corresponding to the periphery of the visual field (see Fig. 6). These findings are consistent with electrophysiological studies in monkeys, showing that the gain of neurons with receptive fields in the periphery of the visual field (>5°), increases when the receptive fields are located in the straight-ahead direction (Durand et al. 2010). We found this straight-ahead bias for peripheral regions in V1 and V2, at about 6° of eccentricity. This may explain why this tuning has not been found in previous fMRI studies, since eye modulations have not been investigated at such eccentric retinotopic regions (Andersson et al. 2007; Merriam et al. 2013). It has been proposed that enhanced responses to a stimulus centered with respect to the head could facilitate efficient navigation around obstacles when gaze is directed toward the periphery (Durand et al. 2010). Behavioral studies suggest that these electrophysiological effects are related to a decrease in detection thresholds and reaction times for objects presented in the straight-ahead direction in comparison with more eccentric targets (Camors and Durand 2011; Durand et al. 2012).

Our results are consistent with idea that gain fields can be described by a planar function of eye positions, how it has been shown in single neurons (Zipser and Andersen 1988; Andersen et al. 1990) and recently with pattern of voxels (Merriam et al. 2013). As shown in Fig. 5, for each region the voxel amplitude had a linear trend with two eye positions that have the highest and the lowest amplitude and the intermediate position (gaze-center) falling between these two cases. Even though the experiment was not meant to test the azimuth dimension, we repeated the same peak analysis on regions near the horizontal meridian (see Fig. 10, Supplementary) and we observed an analogous linear trend, consistently with the idea that gain fields are characterized by a planar function.

### Gaze position affects the BOLD amplitude of retinotopic responses

Our results are consistent with studies in monkeys and humans that show that gaze position does not affect the position of a neuron’s receptive field but does change its response gain (Blohm and Crawford 2009).

We observed no consistent change in the topography of polar angle maps with eye position but did observe significant changes in the BOLD amplitude of the retinotopic responses (Merriam et al. 2013). Gaze direction modulated BOLD responses by as much as 25 %. This figure is in line with results from previous studies in monkeys (Durand et al. 2010), in which neuronal evoked activity had a median increase of 20–40 % when the receptive field was in the center of the visual field relative to the head, compared to a deviation of 10° to the left or right. Our results are consistent with many studies in monkeys showing that gaze position changes the response gain of neurons, but not the retinotopic position of their receptive fields (Andersen et al. 1985; Trotter et al. 1992; Galletti and Battaglini 1989; Galletti et al. 1995; Trotter and Celebrini 1999; Rosenbluth and Allman 2002; Durand et al. 2010).

Our gain modulations cannot be explained by a shift in the retinotopy. We did not monitor eye movement but it is unlikely that eye movements occurred. The subjects were trained psychophysical observers and the reliability of the maps observed here (e.g., discrete mapping of the foveal representation) confirms that subjects maintained a stable fixation during the visual stimulation.

One important issue is the effect of the retinal disparity difference between the upper and the lower field edges, as a function of gaze position (Andersson et al. 2004): in our wide-field display the screen edges are farther from the eyes than the screen center, and as a consequence the image is distorted differently. However, our maximal disparity difference was 1.0°, or approximately 10 % of the stimulus size. This deviation is substantially smaller than observed changes in the BOLD response between gaze-up, gaze center, and gaze-down conditions (Figs. 2, 4, 5, 6). Another possible confound is that the differential BOLD activity reflected effects of distance on perceived size: distant objects that look bigger than identical objects closer to the observer have been claimed to activate a larger area in V1 (Murray et al. 2006; Fang et al. 2009). In our experiment, however, more peripheral portions of the polar angle stimuli, in relation to the subject, activated V1 and V2 less than closer portions of the same stimuli. Another possible source of artifact is related with the luminance of the stimulus display. In eye position experiments it is important that projected stimuli have an identical luminance across the display and that there are no position-independent differences. In this regards, a critical area is the edge of the screen, where LCD projectors might generate a low level of light, thus creating a luminance boundary. To address this issue, we took luminance measures from within the scanner with a fiber-optic connected with either a digital or an analogical luminance meter. We took measures in 24 different display locations corresponding to the area subtended by the polar angle in the three different gaze positions along the vertical meridian. The background for both the retinotopic and gain field experiment was a uniform gray 128 with a luminance of 45 cd/m^2^ in all the measured locations. Both the polar and the eccentricity had an average luminance of 105 cd/m^2^ (min 35 cd/m^2^, max 175 cd/m^2^). As a consequence, possible distortions in the projector do not explain the eye modulations we observed here. Another concern might come from some possible artifacts at the edge of the stimulus, as the wedge has a high contrast in relation to the gray background. However, we minimized this possible confound by selecting ROIs at about 6° of eccentricity, that corresponds at about the center of the activation, so distant from the edges of the stimulus and the screen display. Finally, another possible confound might come from using single-voxel regions, which signal may be small and noisy. However, we also analyzed small regions of interest consisting of about ten voxels averaged along the eccentricity axis, but found a similar effect compared to using single-voxels. Moreover, all our single-voxel regions were selected in the grey matter, in particular on the individual surface of each subject, near the center of the response. These voxels show the greatest task effect relative to their variance. Thus, the gain field modulation that we observed is unlikely to be the result of experimental confounds resulting from the wide-field set-up and the retinotopic stimuli.

### Mechanisms underlying gain field modulations

Gaze position modulations may be mediated by a variety of extraretinal signals. For example, modulations might reflect the integration of proprioceptive signals from ocular muscles, motor efference copy or both (Buisseret and Maffei 1977; Wang et al. 2007). Gaze position modulations might also reflect a bias in the location of spatial attention (Kastner and Ungerleider 2000; Corbetta and Shulman 2002) toward straight-ahead, which would account for the effects of distance from straight-ahead.

A recent paper reported that when gaze position was manipulated, a detection advantage for peripheral stimuli in the straight-ahead direction was maintained under conditions in which subjects simultaneously needed to detect brief dimming of the fixation point (Durand et al. 2012). The authors noted that this result suggested that the straight-ahead bias does not require full attentional resources but otherwise does not rule out an attentional explanation. Qualitatively, we found a similar straight-ahead bias during passive viewing scans and letterotopy scans, but this result also does not provide strong evidence against an attentional interpretation. Future experiments, in which the level of arousal is controlled and subjects are involved in more demanding attention tasks, are required to understand which process is driving this bias for the straight-ahead.

In conclusion, our study reveals that BOLD responses to a stimulus that activates a fixed peripheral retinotopic locus in human early visual cortex, show gaze-dependent modulations in line with recent electrophysiological studies (Durand et al. 2010). Gain modulations reflect the distance of the stimulus from the straight-ahead direction near the vertical meridian and are consistent with a systematic non uniform distribution of eye position neurons.

We clearly show that early visual areas codify stimulus location with a retinotopic coordinates system even though eyes change position. But we also show that retinotopic maps may be significantly modulated by eye position gain fields. Future fMRI studies will be required for investigating the neural mechanism underlying these gains modulations and whether they occur also along the azimuth dimension at such eccentric retinotopic locations. Moreover, another important issue will be to determine, with the appropriate paradigm, whether this tuning for the straight-ahead is present also in higher-order ventral and dorsal areas, particularly in dorsal areas V6 (Pitzalis et al. 2006) and V6A (Pitzalis et al. 2013).

## Electronic supplementary material

Below is the link to the electronic supplementary material.

Supplementary material 1 (PDF 280 kb)
