# Supplementary material for: Eye position modulates retinotopic responses in early visual areas: a bias for the straight-ahead direction
Source: Brain Struct Funct. 2014 Jun 19;220(5):2587–601. doi: 10.1007/s00429-014-0808-7 (PMC4549389; doi:10.1007/s00429-014-0808-7)
Supplement: Supplementary file 1 — Supplementary material 1 (PDF 280 kb) [file 429_2014_808_MOESM1_ESM.pdf]

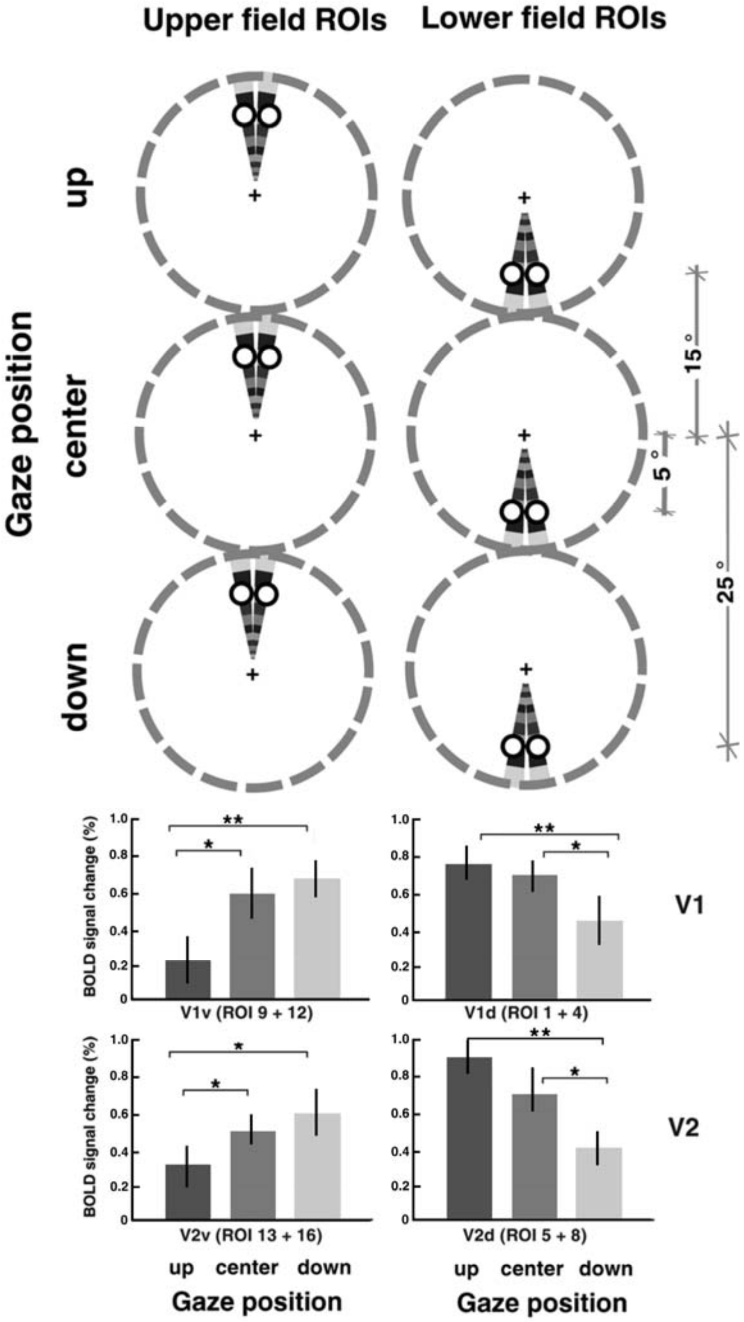

Figure 8 Averaged response amplitudes during gaze-up, gaze-center, and gaze-down conditions in the upper and lower field ROIs.

The vertically aligned dashed circles in each column represent the three different gaze position conditions (-20, 0, +20 degrees vertical). Visual stimulation consisted of a flickering checkerboard wedge rotating in a counterclockwise direction. White points on the wedges indicate the (single-voxel size) regions of interest. For each visual cortical area four regions have been sampled near the

vertical meridian (V1: region 1, 4, 9, 12; V2: region 5, 8, 13, 16; see Fig. 2 in the main text for further ROIs details). Graph bars indicate extracted BOLD responses in the gaze-up, gaze-center, and gaze-down conditions in cortical visual areas V1 and V2. Beta-weights were estimated using the general linear model (GLM) in each of the ROIs for each of the regressors: gaze-up, gaze-center, and gaze-down. The two panels represent the set of points sampled close to the vertical meridian in the upper and lower visual field respectively. Error bars represent ± mean squared error, (* $p < 0.05$, ** $p < 0.01$).

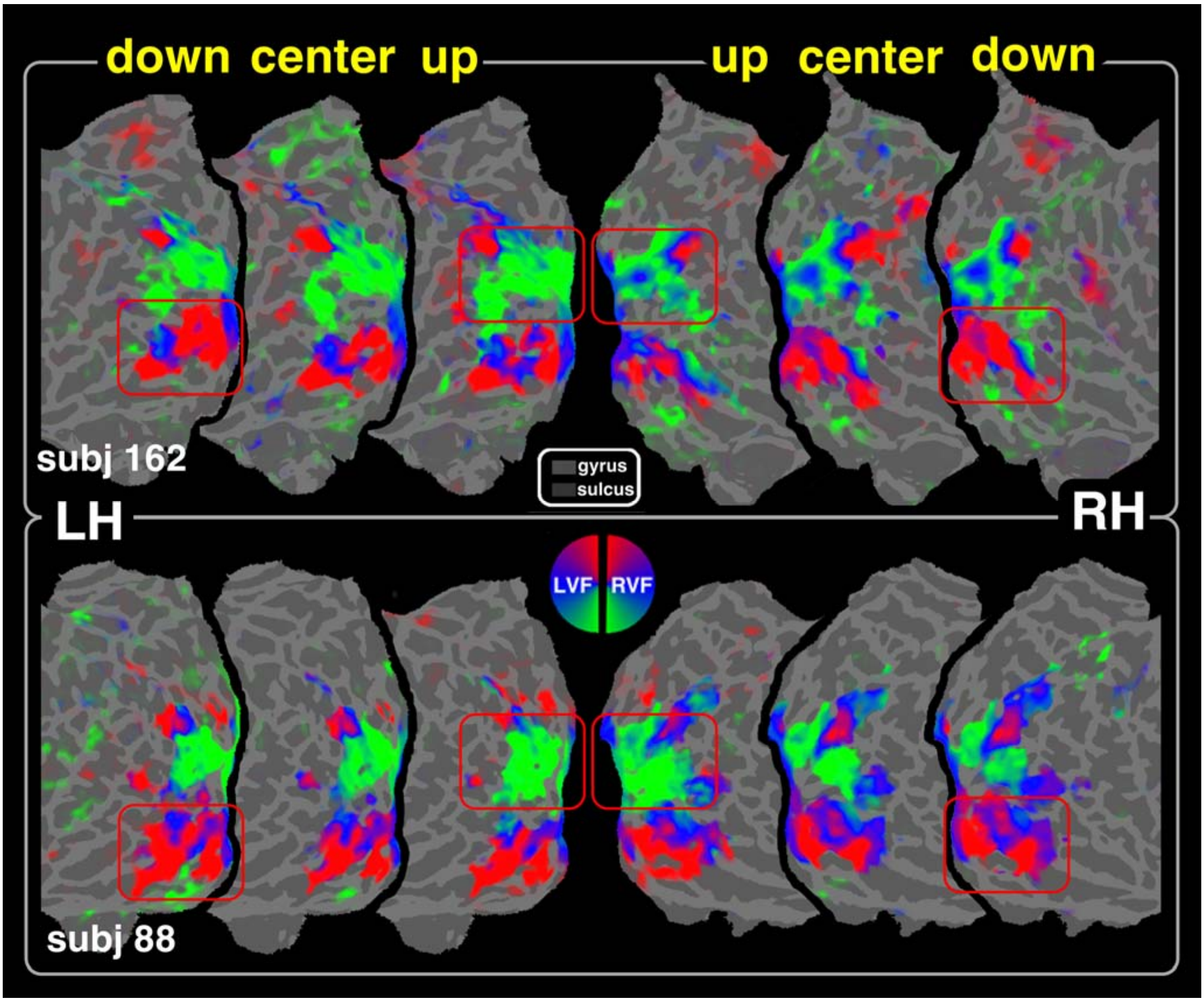

Figure 9 Maps of polar angle in two representative subjects

Colors on the flattened maps codify the response phase of each voxel for a counter-clockwise rotating wedge. Each hemisphere represents the response phase for a particular eye position (down/center/up). Red rectangles underlie enhanced visual areas by gain modulations: dorsal stream in gaze-up and ventral stream in gaze-down conditions.

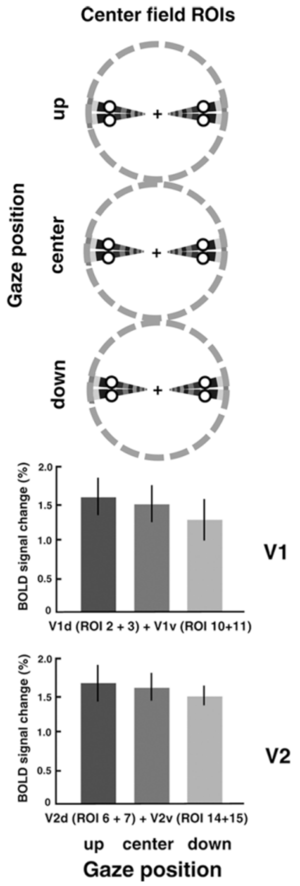

Figure 10 Averaged response amplitude during gaze-up, gaze-center, and gaze-down conditions on the peak response, across all six subjects.

The vertically aligned dashed circles represent the three different gaze position conditions (+20, 0, -20 degrees vertical). White points on the wedges indicate the (single-voxel size) regions of interest. For each visual cortical area four regions have been sampled near the horizontal meridian (V1: region 2, 3, 10, 11; V2: region 5, 6, 14, 15; see Fig. 2 for further ROIs details). Graph bars indicate BOLD signal change in the Gaze-Up, Gaze-Center, and Gaze-Down conditions in cortical visual areas V1 (dorsal and ventral) and V2 (dorsal and ventral). Error bars represent ± SEM.
